# Supplementary material for: Insights into the Synthesis Parameters Effects on the Structural, Morphological, and Magnetic Properties of Copper Oxide Nanoparticles
Source: Materials (Basel). 2023 Apr 27;16(9):3426. doi: 10.3390/ma16093426 (PMC10180341; doi:10.3390/ma16093426)
Supplement: Supplementary file 1 [file materials-16-03426-s001.zip › materials-2342925-supplementary.pdf]

Insights into the Synthesis Parameters Effects on the Structural, Morphological, and Magnetic Properties of Copper Oxide Nanoparticles

Fatma Mbarek [1,*], Ichraf Chérif [1,2], Amira Chérif [3], José María Alonso [4,5], Irene Morales [4], Patricia de la Presa [4,6] and Salah Ammar [1]

1. Electrochemistry, Materials and Environment Research Unit, UREME (UR17ES45), Faculty of Sciences of Gabes, University of Gabes, Gabes 6072, Tunisia; cherif.ichraf@yahoo.fr (I.C.); salah.ammar@fsg.rnu.tn (S.A.)
2. Higher Institute of Education and Continuous Training of Tunis, Virtual University of Tunis, Tunis 1073, Tunisia
3. University of Tunis El Manar, Faculty of Sciences of Tunis, Laboratory of Materials Organization and Properties, Tunis 2092, Tunisia; cherifamira3008@gmail.com
4. Institute of Applied Magnetism, Complutense University of Madrid, A6 22,500 Km, 28230 Las Rozas, Spain; jm.a.r.0@csic.es (J.M.A.); irenemorales@ucm.es (I.M.); pmpresa@ucm.es (P.D.l.P.)
5. Institute of Materials Sciences of Madrid, CSIC, Sor Juana Ines de la Cruz 3, 28049 Madrid
6. Department of Material Physics, Complutense University of Madrid, Plaza de la Ciencia 1, 28040 Madrid, Spain

* Correspondence: fatma.mbarek@fsg.rnu.tn; Tel.: +216-50683270

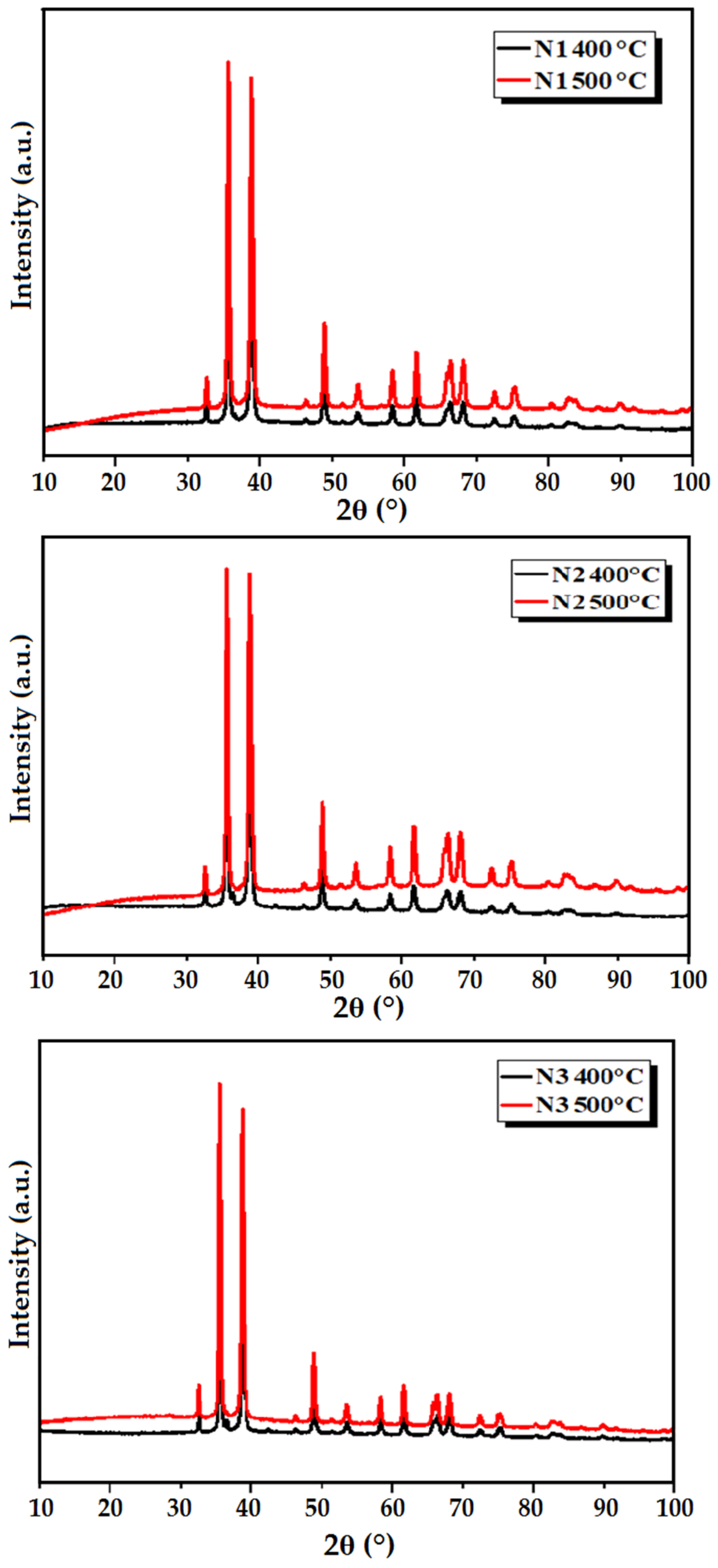

Figure S1. XRPD patterns for N1, N2, and N3 samples synthesized at 400 and 500 °C.
